# Validation of ethnopharmacological uses of *Murraya paniculata* in disorders of diarrhea, asthma and hypertension

**DOI:** 10.1186/s12906-015-0837-7

**Published:** 2015-09-09

**Authors:** Fatima Saqib, Mobeen Ghulam Ahmed, Khalid Hussain Janbaz, Saikat Dewanjee, Hawa ZE Jaafar, Muhammad Zia-Ul-Haq

**Affiliations:** Faculty of Pharmacy, Bahauddin Zakariya University, Multan, Pakistan; Advanced Pharmacognosy Research Laboratory, Department of Pharmaceutical Technology, Jadavpur University, Kolkata, 700032 India; Department of Crop Science, Faculty of Agriculture, University Putra Malaysia, Selangor, Malaysia; The Patent Office, Karachi, Pakistan

**Keywords:** *Murraya paniculata*, Anti-spasmodic, Bronchodilator, Vasodilator

## Abstract

**Background:**

*Murraya paniculata* is traditionally used for management of gut, air way and cardiovascular disorders. The study was conducted for provision of pharmacological rationalization for folkloric uses of *Murraya paniculata* in gut, air way and cardiovascular problems.

**Methods:**

Aqueous-ethanolic extract of Mp.Cr was tested using *in vitro* techniques on isolated tissue of rabbit (jejunum, trachea and aorta) to detect the possible presence of spasmolytic activity. The responses of tissues were recorded using isotonic transducers coupled with PowerLab data acquisition system.

**Results:**

Application of the extract of Mp.Cr relaxed spontaneous and high K^+^ (80mM)-induced contraction in rabbit jejunum preparation. Because it shifted the CRCs (Calcium response curve) towards the right side so the possible blockade was of calcium channel similar to verapamil. In rabbit trachea, extract of Mp.Cr produced relaxation of carbachol and high K^+^ induced contractions. When plant extract was checked further on isolated aorta for its possible vasodilator effect, it caused relaxation of phenylephrine and high K^+^-induced spastic contractions at different doses.

**Conclusion:**

These results indicate that *Murraya paniculata* shows anti-spasmodic, bronchodilator and vasodilator activity facilitated through Ca^++^ antagonist mechanisms.

## Background

The concept of medicinal foods and nutraceuticals is gearing momentum and people are seeking foods having health-promoting and immunity-boosting effects. Markets are now booming with functional foods in various forms and at various prices. Local communities in developing countries use many indigenous foods items as medicinal food without knowing the scientific basis of their healthy effects. Pakistan is known as land of spices and many indigenous spices are being used as medicinal food here. *Murraya paniculata* L. (*Rutaceae*) locally known as *Orange Jessamine* is a commonly used spice in Pakistan. It is added to food and beverages by local peoples to enhance flavor and fragrance besides its various therapeutic applications. Its leaves are used in preparing soup, fish, meat and chicken dishes. The ground bark of stem is used in various drinks while ground root is also eaten.

The plant is well-known due to its therapeutic efficacy also. The ground bark of stem is used as antidote in snake bites while ground root is used to cure body ache. The leaves are stimulant, astringent and utilized by the local community for relief from diarrhea and dysentery [1–3]. It is also used to treat cough, hysteria and rheumatism [4]. It is taken as drink for the treatment of venom bite or as a scrubber on bitted limb. The root and bark is chewed and rubbed to skin to cure body aches. The crushed leaf is applied on fresh cuts, and drunk in dropsy as remedy. It can be used in treatment of toothache, stomachache and gout. It has abortive function and used in treatment of venereal disease [5–7].

Various biological activities of *Murraya paniculata* have been proved like analgesic [8], anti-giardial [9], anti-amoebic [10], antidiarrhoeal, anti-inflammatory, larvicidal, antioxidant, anti-implantation, anti-diabetic, antinociceptive, oxytocic and antifungal activities [11].

*Murraya paniculata* comprises of indole alkaloid, oxygenated flavones, leaves produce oil which contain sesquiterpenes (lcadinene), methyl anthranilate and sesquiterpene alcohol. The oil also contains methylsalicylate, α- cubebene , β-cubebene, β-cyclocitral , isogermacrene, trans-nerolidol, (-)-cubenol, β-caryophyllene, germacrene D and bicycle-germacrene [12].

Although the plant has folkloric repute to manage gastrointestinal, respiratory and cardiovascular diseases but no scientific study has been conducted so far to provide basis for its traditional use. The current study was performed to assess the biological effects of *Murraya paniculata* in diarrhea, asthma and hypertension.

## Methods

### Plant material and extraction

*Murraya paniculata* L. was collected from GC University, Faisalabad in October, 2012 and was authenticated by taxonomist Dr. Altaf Ahmad Dasti of Institute of pure and Applied Biology, Bahauddin Zakariya University, Multan (voucher number STW 439). Plant material was cleared from debris and adulterants, dried in shade for four weeks at room temperature and coarsely ground to powder by special herbal grinder. The ground plant material was saturated three times in order to get the extract. Approximately 1.00 kg of powdered plant material was dripped with aqueous-ethanolic mixture (70 % ethanol + 30 % water) at room temperature in air tight glass jars for 10 to 12 days with random shaking. Organic wastage was segregated by percolating through muslin cloth. The obtained liquid was again filtered through whatman.no 1 filter paper. This process was repeated for three times and the filtrate obtained was combined. The filtrate was evaporated with the help of rotary evaporator (Rotavapor) under reduced pressure at 38 °C that converted filtrate into a thick honey like paste of dark brown color , stored at -4° C in air tight jar [13–15].

### Drugs and animals

Phenylephrine (PE), acetylcholine chloride, carbacol (CCh), potassium chloride, ethylene diamine tetra acetic acid (EDTA), verapamil hydrochloride and magnesium chloride were bought from Sigma Chemicals Co. Glucose, sodium dihydrogen phosphate, Calcium chloride, potassium dihydrogen phosphate ,magnesium sulphate, ethanol and sodium bicarbonate were obtained from Merck, Darmstadt, Germany. Sodium hydroxide, sodium chloride and ammonium hydroxide were purchased from BDH Laboratory. All chemicals were of high purity and grade.

Animals (♂/♀) utilized in these experiments were local breed albino rabbits (1.0–1.8 kg). The animals had not any clinically apparent infection and were kept in stainless cage under controlled environment (12:12 light-dark cycle, relative humidity 55 %, 24–25 °C) at animal house of Bahauddin Zakariya University, Multan. Animals were supplied fresh standard food and water. All experiments were performed according to the rules of the Institute of Laboratory Animals Resources, Commission on Life Sciences, National Research Council [16] and authorized by the Ethical Committee of the Bahauddin Zakariya University, Multan (EC/01/2011 dated 16.02.2011).

### Isolated tissue preparation

All studies were accomplished according to protocol as defined earlier [17–21].

### Rabbit jejunum

Plant extract was checked on isolated rabbit jejunum for the presence of spasmolytic activity. Rabbit was killed at its cervical portion, jejunum portion was taken out [17–21], and 2 cm long piece of jejunum was mounted into an isolated tissue bath having Tyrode’s solution (37 ° C) and aired with carbogen (95 % O_2_ : 5 % CO_2_). The constituents and their concentration used for Tyrode’s solution (mM) were: NaCl (136.9) KCl (2.68), NaH_2_PO_4_ (0.42), MgCl_2_ (1.05), glucose 5.55, CaCl_2_ (1.8) and NaHCO_3_ (11.90) and pH 7.4. Preload (1 g) was exerted and kept constant throughout the experiment. Responses of tissue were recorded isotonically with the help of Bioscience transducers; an equilibrium period of at least 30 min was given prior to any drug addition. Isolated jejunum preparations shows rhythmic contraction spontaneously in controlled experimental conditions and allow the analysis of relaxing effect in the absence of agonist [17–21]. Spasmolytic activity was observed by application of dose of extarct in cumulative fashion. The relaxant effect of plant extract was taken as percentage difference in spontaneous contraction of isolated jejunum preparation noted immediately prior to adding any test material.

### Mechanism of Calcium channel blockade

To confirm the presence of calcium antagonism as most plausible mechanism of action for anti-spasmodic activity, plant extract was tested by relaxation of the sustained spastic contractions after exposure to high concentration of K^+^(80mM) and low K^+^(25mM) used to depolarize the isolated jejunum [22]. Extract was added in cumulative manner to achieve concentration dependent inhibitory responses [23]. The resultant relaxing effect of test material was expressed as percentage of control contractile response that was provoked by high K^+^(80mM) and low K^+^(25mM).

Calcium antagonistic act of plant extract was confirmed by methods reported earlier [17–21]. Isolated rabbit jejunum was stabilized in Tyrode normal (t^n^) solution that was exchanged with Ca^+2^ free Tyrode’s solution for 30 min because EDTA (0.1mM) was added to remove calcium from tissue. Tissue bath solution was then replaced by potassium rich Tyrode’s solution i.e. Calcium free solution consisted of following composition (mM): KCl (50), glucose (5.55), NaCl (91.04), NaHCO_3_ (11.90), MgCl_2_ (1.05), NaH_2_PO_4_ (0.42) and EDTA (0.1). After incubation period of 30 min, cumulative Ca^+2^ concentrations were applied to the tissue bath to obtain control calcium dose response curves (DRCs). When control calcium dose response curves (mostly after two cycles) were found superimposable, jejunum preparation was then washed and treated with extract for one hour and then reconstructed DRCs of calcium for testing possible CCB activity. Dose response curves (DRCs) of Ca^+2^ were constructed in the presence of different concentration of test substance in tissue bath. Verapamil hydrochloride was used as a positive control.

### Rabbit trachea

Rabbit trachea was dissected out and transferred to Krebs solution having the following composition (mM): KCI (4.7), KH_2_PO_4_ (1.3), glucose (11.7), MgSO_4_ (1.2), NaCl (118.2), NaHCO_3_ (25.0) and CaCl_2_ (2.5) at pH 7.4. Trachea was cleaned free from extra fatty material and rings of 2-3 mm width were cut from ventral side in front of smooth muscles layers. These tracheal preparations were mounted in organ bath having Krebs solution kept at 37 °C and aired with carbogen. A resting tension of 2g was provided as preload during whole experiment. Tracheal tissue was stabilized for one hour before application of any drug. Tracheal strip was equilibrated by repeated application of carbacol (1 μM). The carbacol (1 μM) and K^+^ (80 mM) were utilized for production of persistent contractions and then subsequently used for testing of test substances. The bronchodilator action of plant extract was checked by addition of dose in cumulative manner. Isometric responses of trachea were measured by power lab. The standard drugs with Ca^+2^ channel blocking effect (verapamil hydrochloride) was tested on high K^+^ (80mM) and carbacol (1 μM) induced spastic contraction for elucidation of possible mechanism.

### Rabbit aorta

To determine the effect on systemic vascular resistance, isolated rabbit aorta ring was used. Rabbit aorta (2–3 mm) was detached and transferred to kreb’s solution. The isolated tissue was then mounted in organ bath having kreb’s solution aerated with carbogen, at 37 °C. A resting tension of 2–5 g wass applied to tissue and allowed to stabilize for1 h, prior to addition of plant extract. High K^+^ or Phenylephrine (1 μM) was used to induce contractions. Vasodilator effects of plant material were investigated by adding in cumulative fashion in isolated tissue organ bath. Changes in tension of aorta were measured by isometric transducer.

### Statistical analysis

All the results are expressed as mean ± standard error of mean (S.E.M., *n* = number of experiments) and the median effective concentration (EC_50_) with 95 % confidence interval (CI). The dose-response curves were analyzed by non-linear regression software (Graph PAD Prism, Version 5 , San Diego, CA, USA).

## Results

### Effect on jejunum

**Fig. 1:**
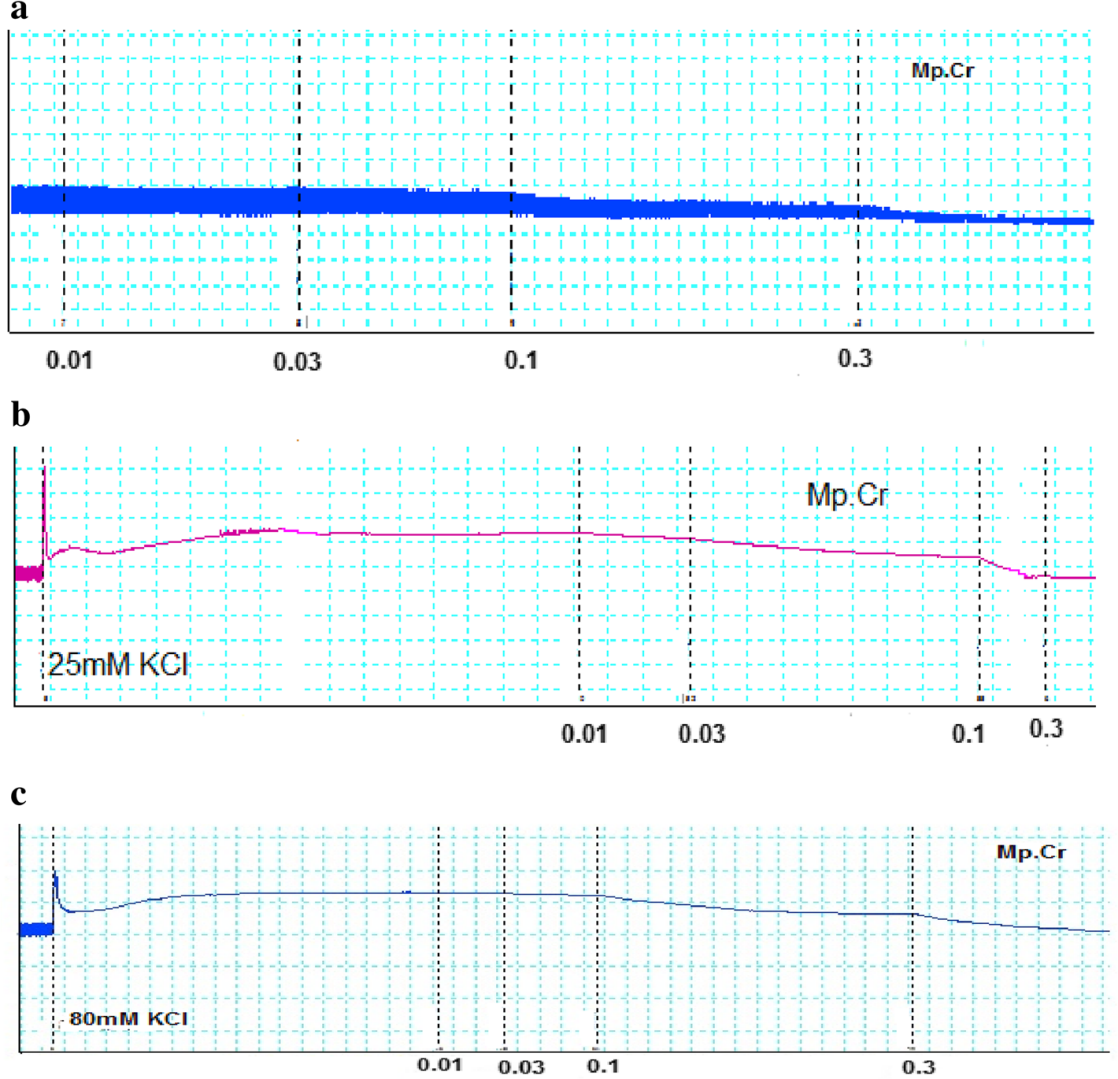
Effect of Mp.Cr on (**a**) spontaneous contractile response (**b**) K^+^ (25mM) induced and (**c**) high K^+^ (80mM) inducing contraction on isolated rabbit jejunum preparation

**Fig. 2:**
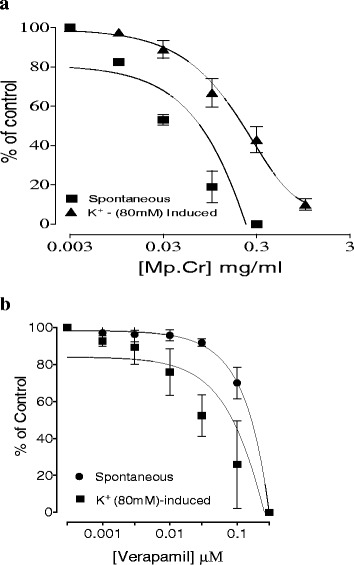
Effect of (**a**) Mp.Cr and (**b**) verapamil on spontaneous and high K^+^ (80 mM) inducing contraction on isolated jejunum preparations of rabbit (values are showed as mean ± SEM., *n* = 5)

**Fig. 3:**
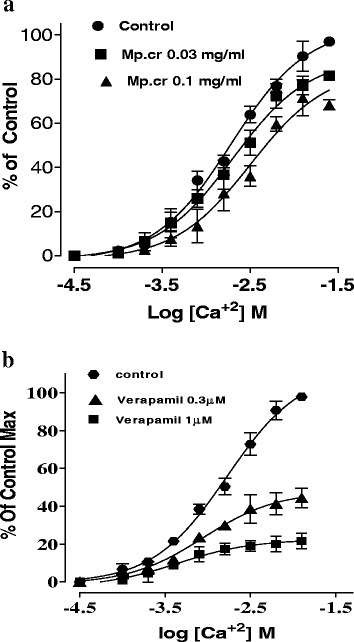
Effect of (**a**) Mp.Cr and (**b**) verapamil on concentration-response curve of Ca^++^ on isolated jejunum of rabbit (values are showed as mean ± SEM., *n* = 3)

Mp.Cr caused inhibition of spontaneous contraction of jejunum, producing spasmolytic effect at a dose range of 0.01–0.3 mg/ml with EC_50_ 0.03610 mg/ml (95 % Cl: 0.02200–0.05923, *n* = 5). Mp.Cr caused relaxant effect on low K^+^ (25 mM) induced spastic contraction (0.3 mg/ml) jejunum preparation whereas it caused a complete relaxation of high K^+^ (80 mM) induced contraction (0.3–1.0 mg/ml) with EC_50_ 0.2723 mg/ml (95 % Cl: 0.1526–0.4859, *n* = 4–5). Verapamil relaxed spontaneous and high K^+^ (80 mM) induced contraction with EC_50_ 0.03446μM (95 % Cl: 0.01956-0.06069, *n* = 5), 0.08421 μM (95 % Cl: 0.04959–0.1430, *n* = 4–5) respectively (Figs. 1 and 2). In addition, pretreatment of isolated rabbit jejunum with Mp.Cr (0.01–1 mg/ml) shifted dose response curve of Ca^+2^ towards right side likewise verapamil (Fig. 3).

### Effect on trachea

**Fig. 4:**
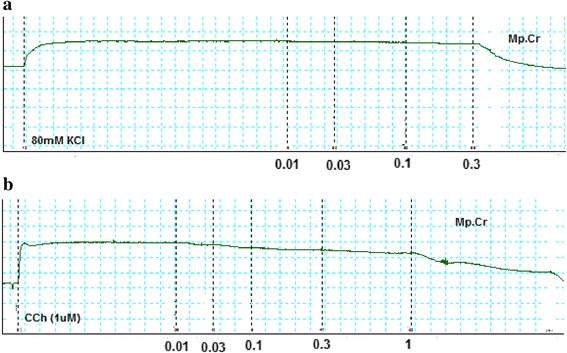
Effect of Mp.Cr on (**a**) K^+^ (80mM)-induced (**b**) Cch(1μM)-induced contractile response on isolated rabbit tracheal preparations

**Fig. 5:**
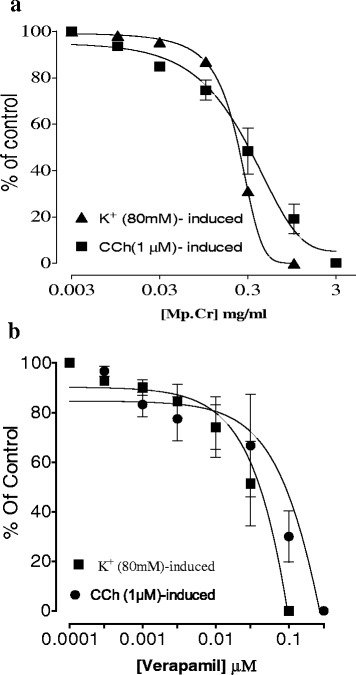
Effect of (**a**) Mp.Cr and (**b**) verapamil on high K^+^ and carbachol (1μM)- inducing contractions on isolated rabbit trachea

The Mp.Cr caused relaxation of K^+^ (80 mM)-induced contraction at 0.3 mg/ml with EC_50_ values of 0.224 mg/ml (95 % Cl:0.1954–0.463, *n* = 4–5) and relaxed CCh (1μM) induced contraction at 1 mg/ml with EC_50_ values of 0.3697μM (95 % Cl:0.2159-0.6332, *n* = 4–5) respectively. Similarly verapamil relaxed K^+^ (80mM) and CCh -induced contraction with EC_50_ 0.114 μM (95 % Cl:0.057–0.227, *n* = 5) and EC_50_ 0.048 μM (95 % Cl:0.028–0.083, *n* = 5) (Figs. 4 and 5).

### Effect on aorta

**Fig. 6:**
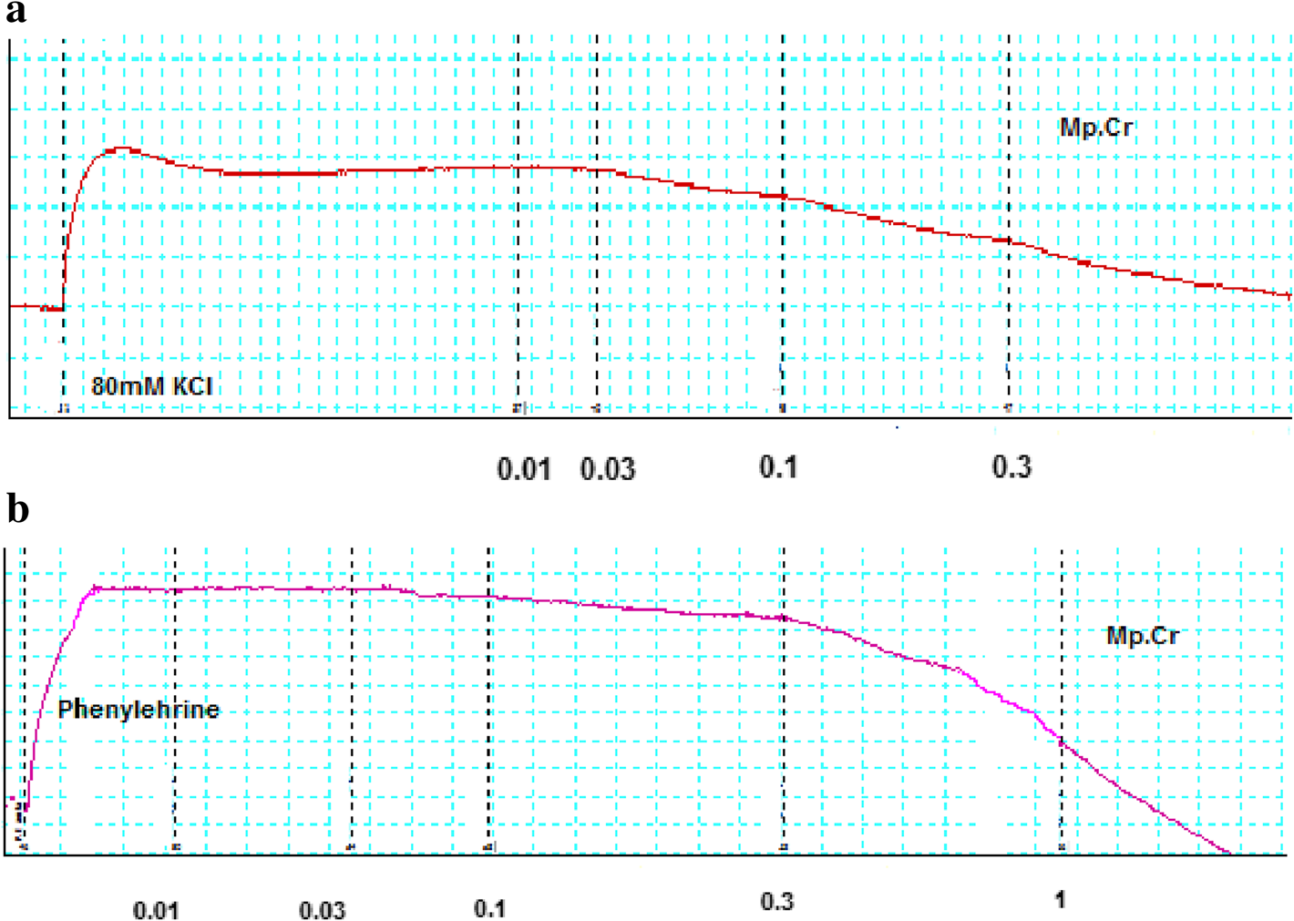
Effect of Mp.Cr on (**a**) K^+^ (80 mM)-induced (**b**) Phenylephrine (1 μM)-induced contractile response on prepared rabbit aortic vascular tissue preparations

**Fig. 7:**
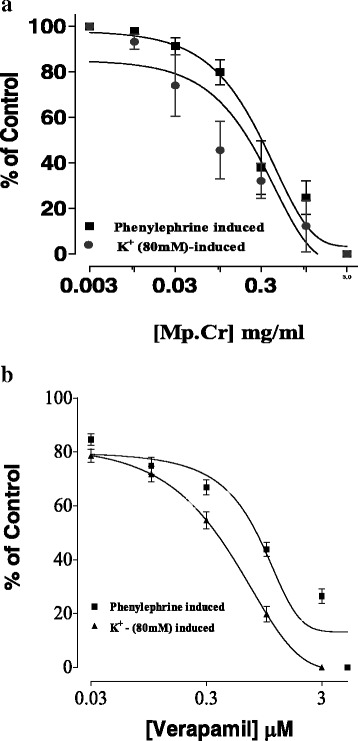
Effect of (**a**) Mp.Cr and (**b**) verapamil on phenylephrine and high K^+^ -induced contraction on isolated rabbit aorta

Mp.Cr caused relaxation of phenylephrine and high K^+^ (80mM)-induced contraction in dose dependent manner with EC_50_ value 0.2894 μM (95 % CI: 0.1633–0.5127, *n* = 5) and 0.08398 mg/ml (95 % CI: 0.05438–0.1297, *n* = 5). Similarly verapamil relaxed phenylephrine and K^+^ (80mM)-induced contraction with EC_50_ 0.90 μM (95 % Cl: 0.032–0.212, *n* = 5) and EC_50_ 0.43 μM (95 % Cl: 0.032–1.98, *n* = 5), (Figs. 6 and 7).

## Discussion

The local communities in developing countries use herbs to cure various diet-related disorders. There is direct relationship between healthy life and plant-based dietary recepies. The bioactive constituents present in these botanicals exert curative and protective effects in various metabolic disorders especially smooth-muscle dependent organs of human body. This study was conducted to provide biological basis for traditional usage of *Murraya paniculata* in gut, airway and vascular disorders.

*Murraya paniculata* showed a significant antispasmodic effect in isolated jejunum experiment. Previous studies showed that plant usually exhibit spasmolytic effect due to calcium channel blocking mechanism or potassium channel opening. When tested on low K^+^ and high potassium (80 mM)- induced spastic contraction on isolated jejunum, the extract produced same relaxation on both spastic contraction. It is well-known that extracts or compounds which relax only low K^+^-induced contractions are potassium channel openers while the extracts or compounds which relax both contraction induced by K^+^ (25mM) and K^+^ (80mM) equally belong to calcium channel blocker. This type of study is also used to distinguish between potassium channel openers and calcium channel blocker [24, 25]. It is interesting that calcium channel blockers are used in management of diarrhea through their anti-spasmodic property.

When KCl is applied on isolated rabbit jejunum preparation, it opens voltage dependent Ca^+2^ channel (VDCs) by the depolarization and Ca^+2^ present outside the cell enters the cytosol [26]. There is interchange between extracellular and intracellular calcium stocks. It is already reported that influx of calcium into sarcoplasmic reticulum through voltage reliant calcium channel causes the repolarization and depolarization of tissues [27–29]. It is not necessary that relaxing effect produced on KCl-induced contraction is due to calcium channel blocking method. To prove assumed mechanism of action of Mp.Cr, calcium dose response curve were constructed in the presence of calcium free medium, the plant extract shifted the curves towards right side similar to that of Verapamil [30]. It is therefore concluded that calcium channel blocking activity is the most probable mechanism of action for anti-spasmodic action.

Calcium channel blockers are useful in asthma and respiratory diseases. Since *Muraya paniculata* is famous for its folkloric use in bronchitis and as decongestant so Mp.Cr was tested on isolated rabbit tracheal tissue preparation. It produced relaxation of tracheal contraction induced by carbachol and high K^+^. Mp.Cr inhibited high K^+^-induced contraction at low dose as compared to CCh -induced contractions in trachea, so it has bronchodilator effect possibly mediated through calcium channel blockade.

Calcium channel blockers are used in treatment of cardiovascular disorder l [31]. The plant extract was investigated for potential vasodilation action as it has folkloric use of dilation of arteries in traditional remedies. It relaxed contractions induced by phenylephrine (1 μM) and high K^+^ on different concentrations of 3 mg/ml and 1 mg/ml respectively. It means that it relaxes high K^+^-induced contractions at lower dose so calcium channel blocking may be possible mechanism for vasodilation likewise verapamil.

## Conclusion

This study indicate that *Murraya paniculata* possesses anti-spasmodic, bronchodilator and vasodilator effect mediated possibly through Ca^++^ antagonist property, which provides pharmacological basis for its folkloric use in hyperactive gastrointestinal and respiratory disorders.
